# Food choice mimicry on a large university campus

**DOI:** 10.1093/pnasnexus/pgae517

**Published:** 2024-11-16

**Authors:** Kristina Gligorić, Arnaud Chiolero, Emre Kıcıman, Ryen W White, Eric Horvitz, Robert West

**Affiliations:** Computer Science Department, Stanford University, Stanford, CA 94305, USA; Population Health Laboratory (#PopHealthLab), University of Fribourg, 1700 Fribourg, Switzerland; Institute of Primary Health Care (BIHAM), University of Bern, 3012 Bern, Switzerland; School of Population and Global Health, McGill University, Montreal, Quebec H3A 0G4, Canada; Microsoft Research, Redmond, WA 98052, USA; Microsoft Research, Redmond, WA 98052, USA; Microsoft Research, Redmond, WA 98052, USA; School of Computer and Communication Sciences, EPFL, 1015 Lausanne, Switzerland

**Keywords:** food choice, campus, social influence, health, sustainability

## Abstract

Social influence is a strong determinant of food consumption, which in turn influences the environment and health. Purchasing mimicry, a phenomenon where a person copies another person’s purchases, has been identified as the key governing mechanism. Although consistent observations have been made on the role of purchasing mimicry in driving similarities in food consumption, much less is known about the precise prevalence, the affected subpopulations, and the food types most strongly associated with mimicry effects. Here, we study social influence on food choice through carefully designed causal analyses, leveraging the sequential nature of shop queues on a large university campus. In particular, we consider a large number of adjacent purchases where a focal user immediately follows another user (“partner”) in the checkout queue and both make a purchase. Across food additions purchased during lunchtime together with a meal, we find that the focal user is significantly more likely to purchase the food item when the partner buys the item, vs. when the partner does not, increasing the purchasing probability by 14% in absolute terms, or by 83% in relative terms. The effect is observed across all food types, but largest for condiments. Furthermore, purchasing mimicry is present across age, gender, and status subpopulations, but strongest for students and the youngest. We elucidate the behavioral mechanism of purchasing mimicry, and derive direct implications for interventions improving dietary behaviors on campus, such as facilitating preordering to reduce detrimental interactions.

**Significance Statement**

Social influences shape diets, which in turn influence health and sustainability. Previous work has demonstrated that purchasing mimicry, a phenomenon where one copies another person’s purchases, drives similarities in food consumption. However, much less is known about the precise prevalence, the affected subpopulations, and the food types most strongly associated with mimicry effects. In this work, we design studies leveraging a large dataset of purchases from a university campus. We find strong evidence of purchasing mimicry, present across age, gender, and status subpopulations but is strongest for students and the youngest. The results elucidate the social determinants of purchasing behavior with high granularity and have direct implications for improving diets on campus.

## Introduction

Diet critically affects health outcomes (1). As a consequence, behavioral interventions (2) and policies that promote healthier diets are a public-health priority (3). Since social influence is known to be a strong determinant of food consumption (4), research has explored the potential of social norms for designing public health interventions to change diets (5) e.g. by promoting healthy dietary habits and physical activity (6), losing weight (7), and reducing food waste (8).

In university campus environments in particular, students and staff consume meals regularly and in large quantities, impacting the environment (9) and health (10). Universities therefore provide an opportunity to study food choice, with implications for the general population. Food consumption on campus is particularly consequential since university education coincides with adolescents’ and young adults’ transition into adulthood. During this period, new dietary habits can be formed, and it is a critical period to stay on a healthy track to reduce the risk of chronic diseases, such as obesity, diabetes, cardiovascular diseases, and cancer (11, 12). Since campuses are training and working environments, university food consumption is also an occupational health issue. Therefore, it is necessary to understand factors influencing behaviors in these environments, and understanding factors can, in turn, inform health-related interventions and policies among university students and staff.

A large body of prior work has consistently observed similarities between connected persons in social networks e.g. friends (13) and family (14, 15), in a number of experimental and survey-based studies (16, 17). The food choices of others have been observed to influence food choices (18–21). Particular focus has been placed on unhealthy behaviors and their social influences (22, 23), observing that obesity (24), overeating (25), fast food (26), and alcohol and snack consumption (27, 28) are impacted by social norms. In an on-campus setting, previous work has longitudinally characterized shifts in the healthiness of food choices after acquiring new eating partners (19).

There are numerous mechanisms postulated about how others influence our food consumption, including the processes of information gathering, minimizing regret, social comparison and integration concerns (5). A key identified mechanism of interpersonal influence on eating behavior is *behavioral mimicry*, referring to copying the behavior of others (29, 30). For instance, individuals automatically mimic the gestures and hand movements of others, as an unconscious attempt to make the other individual like them (31). Since eating is often habitual i.e. automatically driven by external cues, unconscious behavioral mimicry is a key interpersonal influence mechanism when eating with others.

Previous studies have shown evidence of mimicry in food consumption: people tend to adjust their intake directly to their eating companions by eating more when others eat more and less when others eat less (32). Previous work has found evidence of purchasing mimicry in real-life cafeteria settings, observing that individuals mirror vegetarian meal (18, 33) and starter (34) choice of others. Previous studies have also found that pairing a participant with an actor (the “confederate”) influences the amount and type (35) of food eaten by the participant and their biting pattern (36) i.e. whether individuals take a bite of their meal in congruence with their eating companion rather than eating at their own pace (37).

However, several key questions remain unanswered: *How precisely prominent is food purchasing mimicry? What foods are the most associated with purchasing mimicry, and what subpopulations are the most affected?* Previous studies have suggested that some food categories, such as starters, could be more susceptible to social modeling than others (34). Nonetheless, it remains unclear what foods are most associated with purchasing mimicry. Estimating the effect across food types is challenging due to prevailing norms—i.e. when almost everyone (34) or almost no one gets an item (33), estimating the causal effect of a rare exposure is difficult. Similarly, while previous work has reported mimicry between both familiar and unfamiliar individuals (34), it is unclear how different on-campus subpopulations (i.e. students vs. staff) might be affected. Answering these questions and identifying the purchasing mimicry effects between food types and subpopulations is key to determining whether and to which extent purchasing mimicry can be leveraged for behavioral interventions modifying shop layouts, social structures, or food availability.

More fine-grained data sources and design paradigms are needed to precisely identify behavioral mimicry and how it varies across foods and subpopulations, since aggregate insights may not reflect the true effect equally well for everyone, and across all the foods. Digital datasets passively capturing food choices of large populations offer new opportunities to answer these questions. However, observational studies leveraging passively collected datasets face limitations due to the presence of confounding factors and biases. In computational analyses of real-world behaviors, it remains challenging to measure and disentangle properties that are relevant in the context of food consumption, such as attributes of the individuals and the environment. Another challenge is homophily (38), people’s tendency to form ties with others similar to themselves to begin with (20, 39, 40). Altogether, causally identifying social factors from passively collected logs is challenging (41, 42).

###  

####  

##### Our approach

The present study addresses the challenges of understanding the role of mimicry in a university campus environment across food types and subpopulations. We leverage a large dataset of shop records that captures the order of food selection and purchasing, allowing us to measure whether early customers influence late customers. In particular, we analyze an anonymized dataset of food purchases made on the EPFL university campus. The data spans from 2010 to 2018 and contains 18 million purchases made with a badge that allows anonymous linking to a person’s purchase history and basic demographics.

**Fig. 1.**
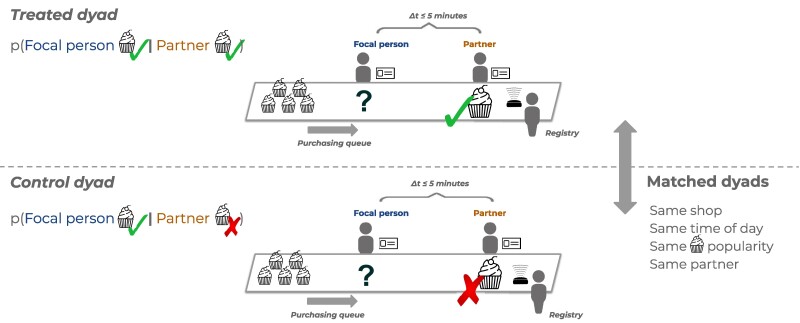
Study design. We identify dyads where two individuals make purchases within five minutes of each other, with no one in between, adjacent in the purchasing queue. The first person to make the transaction in the queue is referred to as the partner, and the second person as the focal person. We are interested in identifying the impact that the purchasing behavior of the partner has on the focal person e.g. purchasing a dessert as in the illustration. To that end, the dyads are matched, such that the dyads are comparable (i.e. they occur in the same shop, time of day, same partner identity, same availability, and popularity of the dessert), but, in treated dyads, the partner purchases a dessert, whereas in the control dyads, the partner does not purchase it. Our study then contrasts the focal person’s probability of purchasing the dessert, given that the partner purchased (treated) or not (control).

Based on the transactional data, we design an observational study to identify and measure mimicry in food purchases. We leverage the sequential nature of shop queues and the fact that, with passively sensed data, we can observe many persons in many situations. We consider a large number of adjacent purchases where a focal user immediately follows another user (“partner”) in the checkout queue and both make a purchase. We identify about 500,000 such *dyads* (adjacent purchases made by the focal–partner pair) (cf. Fig. 1). The large number of dyads, rich data about the environmental context, and information about the individuals’ historical patterns let us make measurements of high granularity and scale.

###  

####  

##### Summary of main findings

Analyzing purchasing behaviors, in line with the existing literature (18, 33, 34), we find significant evidence of mimicry, with partners’ purchases affecting all food types. Across food additions purchased during lunchtime together with a meal, we find that the focal user is significantly more likely to purchase the food item if the partner has already bought the item, vs. when the partner has not. The partner’s choice to purchase an item increases the focal user’s purchasing probability, by 14% in absolute terms, or by 83% in relative terms. Furthermore, we find that this effect diminishes when we measure the influence of a random (rather than directly preceding) partner on a focal user, demonstrating that the observed effect is not an artifact due to other contextual factors (cf. Section ‘Mimicry of partner’s purchases affects all food types’).

The scale of our dataset allows refining existing knowledge on purchasing mimicry across food types and subpopulations. In particular, the largest increase in purchasing probability occurs for condiments, while the smallest occurs for soft drinks. The observed effect is robust across subpopulations and affects all genders and statuses, while it is the strongest for students. Analyzing a much smaller dataset with detailed demographics available, we also find that the effect is the strongest among younger persons (cf. Section ‘Mimicry is strongest among students and the youngest’). Overall, the results of this study elucidate the behavioral mechanism of purchasing mimicry across food types and subpopulations, and have direct implications for the design of policies and interventions, on university campuses and beyond.

## Results

### Study design summary

We leverage a large dataset of shop records made on the EPFL university campus. Each food purchase transaction is attributed with the time it took place, information about the location, the cash register where the transaction took place, and the purchased items. For a subset of users, we additionally leverage demographic information: gender, status at the campus (i.e. whether a person is a student, staff member, or “other” status, such as a visitor), and birth year (statistics about the dataset are outlined in Section ‘Data’). We additionally estimate demographic information for the whole population using the paradigm of amplified asking i.e. by fitting a statistical model to a small subsample with known demographics and applying the model to the remaining population in order to approximately estimate their demographics (Supplementary Material, Section S1.2).

The study design is illustrated in Fig. 1. We identify purchases where individuals make purchases within five minutes of each other, adjacent in the queue, with no one in between (referred to as *dyads*). The first person to make the transaction in the queue is referred to as the partner and the second person as the focal person. We are interested in identifying the impact that the purchasing behavior of the partner has on the focal person i.e. the change in the probability that the focal person will buy a certain food item when the partner buys the same item before the focal person, compared to when the partner does not buy that item. We study dyads where the partner and the focal person are observed together repeatedly (Section ‘Sequential choices: studied dyads’). Note that we have no ground-truth information on whether the partner and the focal person know each other and what their social connection is, if any.

The shops typically open at 7 AM and close at 6 PM. The studied dyads occur during breakfast (6 AM–11 AM), during lunch (11 AM–2.30 PM), or in the afternoon (2.30 PM–8 PM). During the three periods, persons purchase an *anchor*—a meal during lunch or a beverage (coffee or tea) during breakfast or afternoon (Supplementary Material, Fig. S1). In addition to the anchor food item, individuals might purchase an additional item (such as a dessert or a condiment), referred to as an *addition*. In our main analyses, we study the effect of purchasing mimicry of 13 frequent additions (the selection of the food items in focus is outlined in Section ‘Sequential choices: studied dyads’). We ensure that in the dyads the partner and focal person both purchase an anchor item (a meal during lunchtime or a hot beverage during the morning or afternoon/evening), and observe the purchasing of one of the 13 food additions purchased with the anchor.

In this setting, the observed behavior of the focal person is impacted by the partner’s traits through their social tie, and by the partner’s food choice through the sequential ordering in the queue. Additionally, both the partner’s and the focal person’s food choice is influenced by common environmental factors. The setting is informed by standard assumptions made to identify the causal effect of social influence under the presence of homophily in a pairwise setup, when examining the causes behind why a person manifested a behavior at a given time (42, 43). The statistical assumptions and the causal graph reflecting them are detailed in Section ‘Causal assumptions and DAG’ (here ‘directed acyclic graph’ abbreviated as DAG).

The minimum sufficient set of variables to control for (according to the backdoor criteria) are the common environmental factors that day (shop identity, time of day [breakfast time, lunchtime, afternoon], popularity, and availability of the item that day) and the partner’s identity, which captures the partner’s eating profile. In Supplementary Material, Section S1.3, we examine the robustness of our estimates when allowing for violations of these assumptions.

###  

####  

##### Estimation

We then perform matching of dyads such that the dyads are comparable (cf. Section ‘Matched estimation framework’). In the treated dyads in a matched pair, the partner purchases a food item of interest, whereas in the control dyad in the pair, the partner does not purchase the food item of interest. The outcome of the matching is are *matched pairs of dyads*.

After matching, within the matched pairs of comparable dyads where one of the 13 food items is bought or not, we contrast the focal person’s probability of purchasing the food item of interest when the partner purchased the item (treated condition) to the probability when the partner did not purchase the item (control condition). The discrepancy between the two probabilities is then expressed in absolute and relative terms using risk difference (RD) and risk ratio (RR), respectively (Section ‘Matched estimation framework’).

###  

####  

##### Randomized robustness test

Moreover, we consider a randomized robustness test. In each dyad, instead of the partner, we choose a random person from the same queue, on the same day, at the same time of day (breakfast, lunch, dinner). The objective of the randomized baseline is to understand similarities stemming from the contextual factors and not directly caused by the actual ordering of the queue and the partner’s choice. The estimation, as previously described, is then performed by dyad matching after the queue randomization. Note that randomized selection is performed as part of the observational analyses.

### Mimicry of partner’s purchases affects all food types

####  

##### Paired analyses

As a first look into the matched dyads, we test for evidence of purchasing mimicry and aim to identify the effect pooled across food items. The contingency table (Table 1) counts the frequency of the four possible outcomes, comparing matched pairs of dyads where in one dyad the partner buys, and in the other dyad the partner does not buy, the additional food item (e.g. dessert, condiment, fruit, henceforth referred to as an *addition*). Note that the most frequent outcome is that in both matched pairs of dyads, regardless of the partner, both focal users do not buy the addition. The least likely is that in matched pairs of dyads, regardless of the partner, both focal users buy (since purchasing probabilities are in general low, cf. Supplementary Material, Table S1).

**Table 1.** Contingency table.

		¬Partner purchased (control dyad)		Total matched
		¬Focal purchased	Focal purchased	pairs of dyads
**Partner purchased**	**¬Focal purchased**	28,111 (57.97%)	5,221 (10.77%)	33,332 (68.74%)
**(treated dyad)**	**Focal purchased**	12,119 (24.99%)	3,042 (6.27%)	15,161 (31.26%)
**Total matched pairs of dyads**		40,230 (82.96%)	8,263 (17.04%)	48,493 (100%)

The number of matched dyad pairs in each condition (treated and control). In columns, dyads where partner purchased the item, and in rows, matched dyads where partner did not purchase the item.

In particular, the discordant instances among the matched pairs of dyads are informative i.e. the off-diagonal entries in the contingency table, which correspond to matched pairs of dyads where the two focal persons’ purchases differ. If there were no partner effects, the two types of discordant entries would be balanced. However, we observe that focal persons mirror their partners more frequently than they do the opposite (2.3 times more likely). In 25% of matched pairs of dyads, focal persons purchase when partners do and focal persons do not purchase when partners do not. In contrast, the opposite scenario (focal persons doing the opposite of their partners) is rarer, occurring in only 11% of matched pairs of dyads. The imbalance between the discordant instances serves as first evidence of mimicry. Based on the contingency table, we reject the null hypothesis of no treatment effect ($P<10^{-12}$ according to $\chi^{2}$-test of no treatment effect).

###  

####  

##### Risk analyses

Next, pooling the matched pairs of dyads across the different items, we quantify RD and RR (cf. Section ‘Matched estimation framework’), which serve as the main outcomes in our analyses. Overall, across all matched pairs of dyads (13 food item additions e.g. condiment, dessert), we find a RD of 14.22% [13.73%, 14.74%] and a RR of 1.83 [1.79, 1.88], meaning that the partner’s choice to purchase an item increases the focal person’s own purchasing probability by 14.22 percentage points in absolute terms, or by 83.48% in relative terms. In comparison, in the case of the randomized baseline where the purchasing order in the queue is randomized, we find a RD of 1.07% [0.69%, 1.45%] and a RR of 1.07 [1.05, 1.1]. In other words, the partner’s influence on the focal person nearly entirely disappears once the ordering of the queue is randomized. The gap between true and randomized queues is observed consistently across the 9 years spanned by the dataset (Supplementary Material, Fig. S5).

###  

####  

##### Risk analyses across food items

**Fig. 2.**
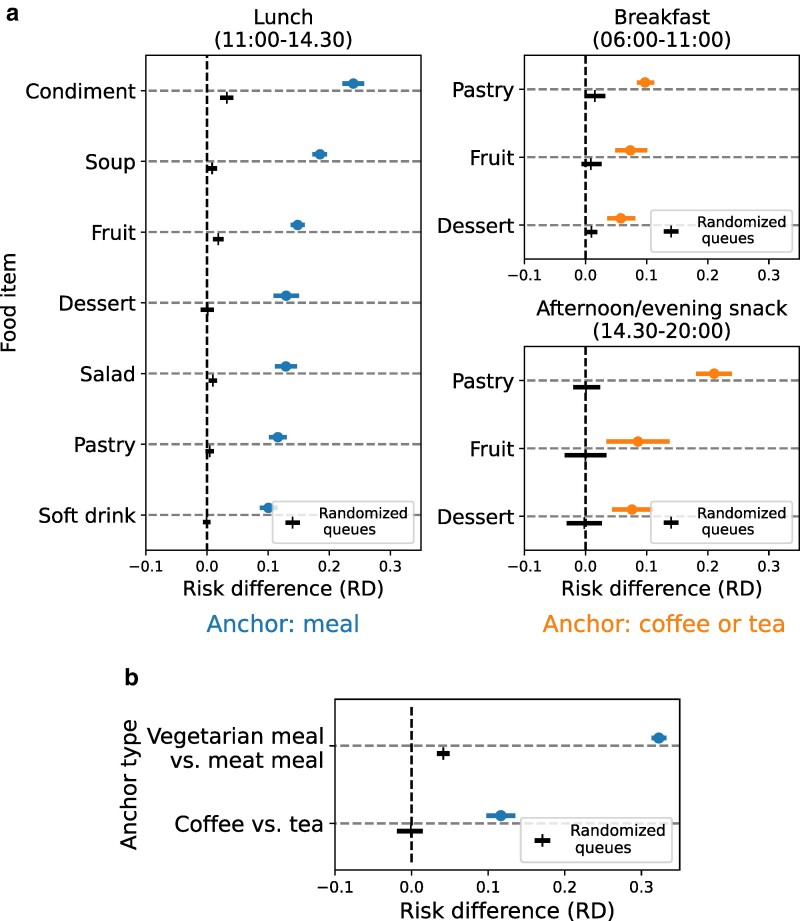
Purchasing mimicry across times of the day and the food items. In a) separately for lunch, breakfast, and afternoon or evening snack, the estimated RD (on the *x*-axis), for the different food item additions (on the *y*-axis). Risk difference estimates are marked with “o” and presented above (on the left: lunch where the anchor is the meal, on the right: breakfast and afternoon or evening snack where the anchor is a beverage). The randomized baseline is maked with “|” and presented below. In b) the estimated risk difference (on the *x*-axis), for the anchor type itself, as opposed to the food item addition (anchor type on the *y*-axis: type of meal, vegetarian vs. not, and type of beverage, coffee vs. tea). The error bars mark 95% bootstrapped CI.

Since effect modification is expected, for the different times of day and across the 13 additions (seven lunch additions, three breakfast additions, three afternoon/evening snack additions), we quantify the RDs separately. We find that all the RDs are significantly different from zero at 95% confidence level (Fig. 2a). The random baseline is much smaller for all additions, among different times of day and among additions. The RD for lunch additions varies between 10.06% [8.65%, 11.42%] for soft drinks and 23.94% [22.11%, 25.76%] for condiments. Risk differences for breakfast additions are 5.78% [3.39%, 8.37%] for dessert, 7.34% for fruit [4.73%, 9.95%], and 9.74% for pastry [8.25%, 11.18%]. For afternoon or evening snack, the RDs are 7.61% for dessert [4.35%, 11.02%], 8.58% fruit [3.85%, 14.16%], and 21.06% for pastry [18.22%, 23.89%]. The relative version of these findings (measured by relative risk) is presented in the Supplementary Material, Fig. S6.

###  

####  

##### Risk analyses across anchors

Although our main analyses focus on food addition items, we also analyze the mimicry of the anchor itself, within the matched pairs of dyads (Fig. 2a). Here the meal anchor comparable dyads can be vegetarian or meat-based, whereas the beverage anchor can be coffee or tea. We observe significant RDs for meal type (32.28% [31.39%, 33.21%]) and beverage type (11.65% [9.72%, 13.52%]). The randomized baseline is again much lower in both cases (meal type: 4.14% [3.30%, 4.95%]; beverage type: 0.10% [−0.02%, 0.01%]). We suspect that mimicry is stronger for the meal-type anchor because purchasing vegetarian food is a behavior related to health and sustainability and, therefore, potentially more likely to be impacted by social norms (44). We also performed a robustness test requiring that the matched pairs of dyads contain exactly the same anchor (meal vs. vegetarian mean; coffee vs. tea), described in the Supplementary Material, Section S1.8 and leading to similar findings as above.

To summarize, among the matched pairs of dyads, we find significant mimicry of partners’ purchases affecting all food types. The partner’s influence on the focal person diminishes once the ordering of the queue is randomized.

### Mimicry is strongest among students and the youngest

####  

#####  

###### Estimated status (students, staff, and other statuses, such as visitors)

**Fig. 3.**
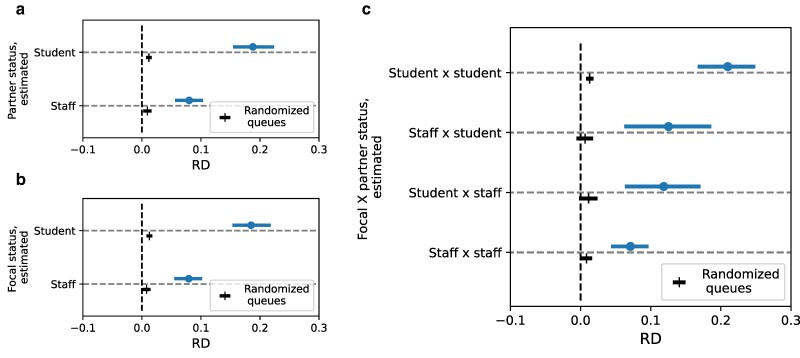
Effect by the estimated status on campus. The estimated RD across the matched pairs of dyads (on the *x*-axis), depending on the individuals’ estimated status (on the *y*-axis). The error bars mark 95% bootstrapped CI. Risk difference estimates are marked with “o” and presented above, while the randomized baseline is marked with “|” and presented below. In a), for partner’s status, in b), for focal person’s status, in c) for the four combinations of the focal–partner status.

We next measure the effect among subsets of matched pairs of dyads based on the estimated status of the partner and the estimated status of the focal person in Fig. 3. We find that the effect is stronger when the partner is a student (RD 16.78% [16.10%, 17.46%]) vs. staff member (10.25% [9.39%, 11.12%]; Fig. 3a). Similarly, the effect is stronger when the focal person is a student (17.0% [16.30%, 17.73%]) vs. staff member (10.01% [9.16%, 10.91%]; Fig. 3b). Examining the four configurations of status within the partner–focal dyad (Fig. 3c), we find that student–student is the condition with the largest risk difference (17.89% [17.11%, 18.60%]). In contrast, the staff–staff condition has the smallest RD (9.66% [8.60%, 10.68%]).

The observation regarding students vs. staff differences holds across the different foods. We measure the RD separately among estimated student–student dyads vs. all nonstudent–student dyads where students can be focal or partner, but not both (Supplementary Material, Fig. S2). We find that across the three times of day and the different food items, the effect is consistently greater among the student–student dyads, implying that the difference depending on the status cannot be explained by discrepancies in preferred food items between students and staff. Instead, known moderators of mimicry, including social, emotional, and personality factors, might vary systematically between students and staff and lead to more or less mimicry (32).

####  

#####  

###### Demographics: true status, age, gender

We next investigate the effect across all the matched pairs of dyads within the subpopulation with ground-truth demographic data (Supplementary Material, Fig. S4). First, among the subpopulation with ground-truth status (as opposed to estimated status, as used above), we consistently find that the effect is stronger both when the partner is a student (10.73% [5.67%, 15.59%]) vs. staff member (5.68% [1.85%, 9.38%]), and when the focal person focal is a student (14.15% [9.56%, 19.11%]) vs. staff member (7.22% [2.44%, 11.38%]). Note that the relative ordering is the same as when using estimated status labels. However, the differences are not statistically significant, likely due to the smaller sample size, relative to the above analysis with estimated status labels.

Second, we investigate the role of age. Given the birth date and the time of the transaction, we calculate the age at the time of the transaction, and we bin the age into terciles. We find that the effect is the strongest when both the partner and the focal person are in the youngest group (up to 22 years old at the transaction time). Examining the partner’s age, we find that the effect monotonically decreases with age (up to 22 years old: 12.04% vs. 23–32 years old: 8.11% vs. over 32 years old: 4.98%), and similarly for the focal person’s age (up to 22 years old: 17.72% vs. 23–32 years old: 13.18% vs. over 32 years old: 4.04%).

Third, regarding gender, we find significant and similar effects among all subpopulations, with a RD greater than zero both when the partner is male and when the partner is female, as well as both when the focal person is male and when the focal person is female. To summarize, food choice mimicry is not restricted to particular subpopulations, but observed across all genders, ages, and statuses. The effect is strongest for student-student dyads and among younger persons (Supplementary Material, Fig. S4).

### Mimicry decreases with time lag

In case of a true causal effect, one would expect a dose–response relationship where the focal person’s purchasing probabilities in the matched pairs of dyads diverge more when the two person in the respective dyad are further apart in the queue, as in such cases the focal person is more likely to have seen the choice of the partner. Hence, we next investigate whether such a dose–response relationship is observed in the data.

We find that, as the distance (measured in seconds) between the dyads in the purchasing queue increases (distribution illustrated in Supplementary Material, Fig. S3), the effect estimate decreases (Supplementary Material, Fig. S3). Note that the distance measured in seconds is calculated for a dyad purchasing at the same registry, meaning that the physical layout of the space and the physical distance between queues are not impacting the temporal distance within a dyad. We measure a significant negative association between the delay in the purchasing queue and RD (the slope of the linear regression β=−0.002, two-sided $p=8.7\times 10^{-5}$) and between the delay in the purchasing queue and RR (β=−0.03, two-sided $P=2.2\times 10^{-6}$). Overall, a larger effect is observed for smaller distances in the queue, such that risk ratio increases by 1.98% every 10 seconds.

If other factors were causing the purchasing similarity, such as a third party present in the shop and convincing individuals to purchase a food item or not, and such factors had nothing to do with the ordering and the distance in the purchasing queue, we would not expect to see a dose–response relationship. The latter thus provides further evidence of a causal effect.

### Robustness tests

####  

##### Sensitivity analysis

Our findings rely on the assumption that there are no unobserved variables creating differences between the matched pairs of dyads that could explain the measured purchasing similarity between partners and focal persons (cf. Section ‘Causal assumptions and DAG’). We perform sensitivity analysis to quantify how the estimates made here would change if this assumption were violated to a limited extent. How strong would the unobserved biases need to be to explain the difference in outcomes between the two sets of matched pairs of dyads? Specifically, we measure the following: if there is a violation of the randomized treatment assignment among the matched pairs of dyads (the choice of the partner), how large would it need to be in order to alter the conclusion that the null hypothesis of no differences depending on focal person’s choice can be rejected? This quantity is quantified with *Γ*, specifying the ratio by which the treatment odds in two matched pairs of dyads would need to differ to result in a *P*-value above the significance threshold (larger values of *Γ* correspond to more robust conclusions).

For P=0.05, we measure sensitivities *Γ* ranging between 1.43 (purchasing a pastry with a breakfast beverage) and 7.22 (purchasing a condiment with lunch). The results of the sensitivity analysis are summarized in Supplementary Material, Fig. S3. Additionally, we perform amplification of the sensitivity analysis (45), where *Γ* is expressed in terms of two parameters *Λ* and *Δ*, as Γ=(ΛΔ+1)/(Λ+Δ). Here, *Δ* is defined as the strength of the relationship between the unobserved covariate and the difference in outcomes within the matched pair, whereas *Λ* is defined as the strength of the relationship between the unobserved covariate and the treatment assignment.

For combinations of *Λ* and *Δ* in the orange area in the figures, significant effects would be detected (leading to P<0.05). In contrast, no significant effects would be detected for the combinations in the blue area (leading to P>0.05). An infinite number of (Λ,Δ) combinations fall on the border. For instance, in the case of purchasing fruit during breakfast, (Λ,Δ)=(5.0,9.8) corresponds to an unobserved covariate that increases the odds of treatment 5-fold and multiplies the odds of a positive pair difference in the outcomes by 9.8. Such amplification is relevant when the concern is not about the violation of randomized treatment assignment but about the presence of specific unobserved covariates with assumed *Λ* or *Δ*. Overall, we conclude that the study design is insensitive to moderate biases (46).

###  

####  

##### Coordination hypothesis

Lastly, we investigated an alternative hypothesis (cf. Supplementary Material Section S1.6) where the observed similarities between dyads are driven by the fact that the two persons coordinated to go for a meal together and agreed on the food choice before lining up in the purchasing queue. In such an alternative scenario, people agree in advance, so the order of how they go does not make a difference. However, since we find that the order of how two persons go in the queue does make a difference, we argue that it does not appear plausible that prepurchase coordination can entirely explain the measured effect.

## Discussion

The results presented here document the prominent role of purchasing mimicry and highlight the need for taking it into account when designing dietary interventions and policymaking around how the foods are offered on university campuses and beyond. First, we find significant mimicry of eating partners’ purchases affecting all food types, in line with the existing literature (18, 33, 34). The partner’s influence on the focal person essentially disappears once the ordering of the queue is randomized (cf. Section ‘Mimicry of partner’s purchases affects all food types’). Second, we find that the effect is not restricted to particular subgroups, but is robust across gender, age, and status groups, with the strongest effect sizes for students and younger persons (cf. Section ‘Mimicry is strongest among students and the youngest’). Finally, we find that food choice mimicry decreases with distance in the purchasing queue following a dose–response relationship (cf. Section ‘Mimicry decreases with time lag’).

Our findings, by relying on observations with a greater statistical power, confirm and refine the existing knowledge about purchasing mimicry. For instance, social influence in dietary habits has previously been examined in the context of school children (16) and adolescents (47–49), who are theorized to be most susceptible to social pressures to diets and activity patterns (50). Although previous experimental studies found relationship type, gender, and age group not to be significant predictors of eating mimicry (35), a recurrent issue faced by previous studies is the small sample size.

We discover the role of age, since we find the effect to be the strongest in the youngest subpopulations and students. We hypothesize that the effect is the strongest among the youngest subpopulations for two potential reasons. First, young people have been documented to be most susceptive to the influence of others due to normative conformity and lowered risk perception (51). Second, younger populations might be more prone to the influence of others due to being novelty-seeking in their dietary behaviors (52). Exploratory analyses corroborate this hypothesis—given the same number of executed transactions, students buy a larger number of different products and visit a larger number of shops than staff members (Supplementary Material, Table S6).

###  

####  

##### Policy implications

The behavioral mechanism of purchasing mimicry has implications for policies and interventions. The fact that we observe discrepancies between subpopulations (e.g. students vs. staff) implies that policymakers should take these differences into account when designing food offering layouts and social interventions. While previous work has focused on the meals (53), our findings imply that availability interventions targeting supplementary food items (such as fruits and desserts, as opposed to meals) may be further amplified by the mechanisms of social norms. In what follows, we outline how insights regarding behavioral mimicry can inform layout, social, and availability interventions.

###  

####  

##### Layout interventions

Future work should determine the effectiveness of interventions that aim to reduce detrimental interactions e.g. shifting the default mode of purchasing by enabling preordering a meal through an application, as opposed to deciding on the spot, since it is known that impulse-buying is mediated by temporal proximity and making decisions in the proximity of others (54). Such interventions should be explored in conjunction with designing dedicated queuing lines to control mixing of people at the check-out registers (e.g. via separate lines for students and staff to modify the opportunities for social influence).

##### Social interventions

Similarly, dietary interventions can involve rethinking the design of queuing systems to increase the likelihood that dyads with specific characteristics appear. For instance, a “bring a student to lunch” day, where a faculty member takes a student for lunch and is reimbursed if they order a healthy meal, might incentivize specific pairings and corresponding queuing sequences to promote purchases of healthy foods. Since the strongest effects are observed for student–student dyads, interventions can incentivize social eating with students who purchase nutritious items, by providing them with vouchers to bring a friend to lunch. Social interventions leveraging influence agents, i.e., selected and trained peers (55), as well as interventions leveraging tools to simulate such agents (56, 57) offer further opportunities to modify behaviors via the social mechanisms.

###  

####  

##### Leveraging mimicry to bolster availability interventions

Lastly, mimicry effects can be leveraged to amplify the impact of point-of-purchase availability intervention strategies (58). To *reduce* purchasing of calorie-dense, low-nutrient foods, the availability of additions is a good opportunity for intervention, since additions are particularly affected by mimicry (strongest effect is measured for condiments, cf. Fig. 2).

We note that such nudges leveraging layouts, social interactions, and availability are not the only possible way of improving nutrition. Instead, structural interventions such as taxes, regulations, and other incentive shifts are likely needed to significantly and permanently change behaviors.

###  

####  

##### Limitations

Our study examines the behavior of a population situated in Switzerland, a large fraction of which is young and not representative of the global population. Also, the individuals in our population do not exclusively consume foods bought on campus. They may bring food to campus from elsewhere, and they also consume food off campus, implying that food purchase behaviors of our population are only partially observed. It is also unknown when the purchasing decision is made, since the purchasing decision is only measured through the logged purchasing act. The robustness rest accessing possible prepurchase coordination and decision-making (cf. Section ‘Robustness tests’) aims to address this limitation. A further source of measurement error is the fact that the estimation of status is imperfect and that individuals with demographic information might not be representative subpopulation of the complete campus population.

Starting from a set of 16.6M transactions executed in a shop and assigned a person ID, we identified 1M transactions paired into 500,000 dyads executed in close temporal proximity, by people who often eat together. This design choice was made with the goal of studying choices made nearby, by frequent partners, in order to be able to repeatedly observe the same individuals and control for their identity, as necessary to isolate the mimicry effect (cf. Section ‘Causal assumptions and DAG’). However, the identified subset of purchases and the individuals that execute them might not be representative of the complete set of transactions and all the individuals on the campus. Those who eat in close temporal proximity to others might be different from those who only visit shops on less busy occasions and might not exhibit the mimicry patterns described here. For instance, they might be more social, younger, and therefore more susceptible to the choices of others (59, 60). Thus, we can only make claims regarding the studied purchase instances and the observed individuals.

Lastly, we do not have access to fine-grained inventory information used for keeping track of items available at shops at a given time. Therefore, we approximate item availability at the purchase point by identifying what items were bought at least once, rather than via explicit availability information. Therefore, the availability at the purchase point can conceivably vary between dyads in ways that cannot be measured from sales logs alone. Further biases stem from the fact that purchasing behavior and choice mimicry might be driven by other unobserved factors e.g. purchasing power, personal relationships, overall health and wellbeing, or calorie need. The threat to validity from such unobserved confounds is mitigated by our sensitivity analysis (cf. Section ‘Robustness tests’), which led us to conclude that the study design is insensitive to small and moderate biases (46).

###  

####  

##### Future work

This study opens the door for future research directions and potential follow-up studies of the social determinants of food choice. Future work should focus on further understanding what drives the differences between age and status. Moreover, our analyses observe dyads only. Future work should study more complex group dynamics beyond dyads that might take place in purchasing queues. Our study focuses on additional food items since information about meal anchors is limited. Future studies should collect rich information about meals and the impact of social interventions on more granular sustainability and nutritional outcomes such as ingredients, nutrients, calories, sustainability metrics (e.g. carbon footprint), and food waste statistics. Finally, future work should determine the extent to which these results generalize beyond university campus environments, to the general population and further settings where people make food choices while exposed to the choices of others.

##### Conclusion

The results of this study elucidate the behavioral mechanism of purchasing mimicry across subpopulations and food types, and have implications for understanding dietary behaviors on campus. Furthermore, we demonstrated how purchase logs can be leveraged to derive insights into social determinants of dietary behaviors. We hope that this study will inspire other institutions to consider analyzing purchase logs collected as part of regular operations in order to derive insights and design interventions with tangible benefits across communities.

## Materials and methods

### Data

We leverage an anonymized dataset of food purchases made on the EPFL university campus. The data span from 2010 to 2018, and contains about 18 million transactions made with a badge that allows linking to an anonymized person’s ID. The data include 38.7 k users who, on median, are observed for 578 days and make 188 transactions. Each transaction is additionally attributed with the time it took place, information about the location, the cash register where the transaction took place, and the purchased items. The data cover all the food outlets permanently located on campus, including restaurants, cafes, and vending machines. We analyze adjacent purchases (referred to as *dyads*) made in one of the 12 major catered shops (as opposed to self-service vending machines i.e. 16.6 M transactions in total). The shops are illustrated in the Supplementary Material, Section S1.7. Furthermore, food items are associated with unstructured textual descriptions. The unstructured textual descriptions were additionally manually mapped to categorical labels (such as “meal” or “dessert”) by a research assistant, who labeled the 500 most frequently purchased items, which account for 95.4% of the total volume of item purchases observed in the dataset. We also rely on a smaller-size enriched transactional dataset gathered during a three-week campus-wide sustainability challenge in November 2018, during which 1,031 consenting participants formed teams to compete in taking sustainable actions. For this subset of users, we leverage demographic information: gender (584 female, 447 male), status at the campus (724 students, 280 staff, 27 other), and birth year (average 1991, median 1994, Q1 1988, Q3 1998).

### Sequential choices: studied dyads

In order to identify purchasing mimicry, we observe a sequence of transactions made using staff or student badges in the queue of a cash registry, in a given shop. We identify instances when two individuals are adjacent in the queue and make a transaction within five minutes of each other, with no one between them. We observe two individuals making a purchase sequentially in a purchasing queue with the badge, as illustrated in Fig. 1.

Co-purchasing matrices (Supplementary Material, Tables S3–5) outline the dyad frequency among the subset of the studied dyads with demographic data available. The tables illustrate a preference for eating with others of the same gender, age, and status, reflecting how social identity differences are situated within educational and occupational social networks (61). We also note that the order female-male is more common than the order male-female. Similarly, the order staff-student is more common than the order student–staff, likely reflecting social norms of politeness and giving way to others depending on their gender and seniority.

A unit of analysis is an instance of two persons having a meal together (a dyad), operationalized as two individuals executing transactions consecutively in the same shop, at the same purchasing line, on the same day, within a 5-minute window, with no one else executing a transaction in between. The 5-minute interval was selected since long inter-arrival times are overall rare; 98.2% of dyads have inter-arrival time of under a minute (the inter-arrival time between the two purchases in a dyad is on average 13.9 seconds, STD=16.4second). There are three daily three peaks of transactions. The studied dyads occur during the time of breakfast (6 AM–11 AM), lunch (11 AM–2.30 PM), or afternoon (2.30 PM–8 PM). During the three periods, persons purchase an anchor—a meal during lunch or a beverage (coffee or tea) during breakfast or afternoon (Supplementary Material, Fig. S1). Coffee is a more frequent anchor compared to tea. During breakfast, tea is an anchor in 12.99% of dyads, vs. coffee in 87.01%. During afternoon, tea is an anchor in 14.05% of dyads, vs. coffee in 85.95%.

In addition to the anchor food item, individuals might purchase an additional item (such as a dessert or a condiment), referred to as *an addition*. In our main analyses, we study the effect of purchasing mimicry of the frequent additions. The additions were selected to include all food items where among the dyads with the anchor, in at least 1% of dyads, the partner buys the addition (Supplementary Material, Table S1) (i.e. at least 1% of the dyads is treated). In total, there are three types of additions frequently purchased together with a beverage during breakfast and afternoon hours (fruit, dessert, and pastry), and seven types of additions frequently purchased together with a meal during lunch hours (condiment, salad, pastry, dessert, soup, soft drink, and fruit). Note that pastry is a separate category from dessert since it can be savory.

Overall, we analyze 509,220 identified dyads. All dyads took place in one of the 12 major shops with a served cash-register. The 509,220 dyads are executed by 18,494 unique individuals. The instances are selected such that the two individuals make at least 10 transactions together adjacent in the purchasing queues in order to be able to observe the same pairs repeatedly, at least 10 times. Robustness tests show that the main findings are robust to this choice (Supplementary Material, Section S1.4), indicating that only people who know each other very well buying similar foods is not an alternative explanation for the results. The threshold was selected to be able to repeatedly observe the same individuals and control for their identity, as necessary to isolate the mimicry effect (cf. Section ‘Causal assumptions and DAG’). In a supplementary analysis, we examine the impact that the number of adjacent transactions has on the estimated effect size (Supplementary Material, Section S1.3).

### Causal assumptions and DAG

We are interested in measuring the causal path of social influence by observing the outcomes $Y_{b}(t)$ and $Y_{a}(t)$ across numerous instances where such outcomes are observed. In particular, we are interested in the chances that the observed outcomes contain identical items, due to the theoretical importance of “matching” the social norm and uniformity seeking through behavioral mimicry. We are interested in the causal path of purchasing mimicry i.e. estimating the causal effect of the treatment ($Y_{a}(t)$) on the outcome ($Y_{b}(t)$).

In particular, we observe a person *b* (focal person), choosing items to purchase at time *t*, $Y_{b}(t)$. The focal person’s choice $Y_{b}(t)$ is governed by the focal person’s eating profile $X_{b}$. Additionally, we consider common environmental factors in the specific dyad *t*, P(t), that can influence the choices of both observed individuals. Common environmental factors are operationalized as the location, the time of day, popularity, and availability of the item at the shop on the given day.

Furthermore, positioned in front in the queue, before person *b*, there is a frequent peer, person *a* (partner), choosing items to buy. Similarly, the partner’s eating profile $X_{a}$ impacts their choice $Y_{a}(t)$. Focal person *b* can be influenced by person a (partner) in their food choice $Y_{a}(t)$, corresponding to the causal path of food purchasing mimicry between $Y_{a}(t)$ and $Y_{b}(t)$.

The peer’s choice can influence the observed person’s choice through other biasing paths. In the presence of homophily, the social tie between persons a and b, $S_{a,b}$ is influenced by the traits of each individual $X_{a}$ and $X_{b}$, since more similar people tend to be closer friends given homophily, and in turn, influences the observed behavior $Y_{b}(t)$ through homophilic biasing paths, for closer friendship might make mimicry stronger. Eating profiles composed of habits and preferences are unchanging and independent of individual choices *t*. Social tie strength is a property of the network and is independent of the timing of individual choices *t*.

In other words, we make the following assumptions:

Assumption 1 The traits of partner $X_{a}$ can influence the observed behavior of the focal person $Y_{b}(t)$ only through $S_{a,b}$ (we investigate this assumption further in Supplementary Material, Section S1.3 by considering alternative DAGs).

Assumption 2 We assume that $Y_{a}(t)$ influences $Y_{b}(t)$ through the ordering in the queue, while $Y_{b}(t)$ is not influenced by $Y_{a}(t)$, i.e. no coordination before purchasing (we investigate this assumption further in Supplementary Material, Section S1.6).

Assumption 3 There are no other unobservable biases (we investigate this assumption further in Section ‘Robustness tests’ via sensitivity analysis).

**Fig. 4.**
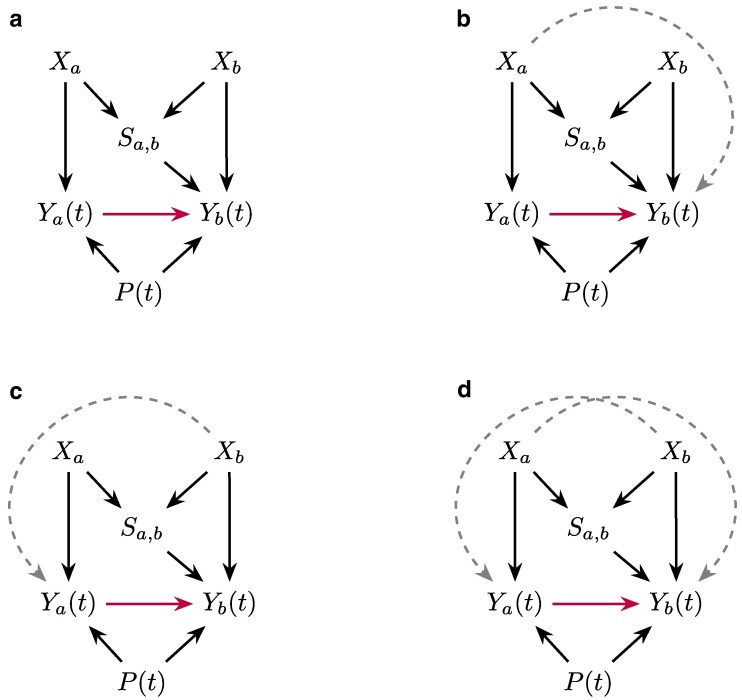
DAGs encode the assumptions about the causal relationship between variables. $X_{a}$ and $X_{b}$ are partner’s and focal person’s eating profile respectively; $S_{a,b}$ is the social tie strength; $Y_{a}(t)$ and $Y_{b}(t)$ are partner’s and focal person’s sets of purchased items at time *t* respectively, and P(t) are common environmental factors at time *t*. Arrow from partner’s to focal person’s sets of purchased items marks the causal path of purchasing mimicry. In a), the assumed DAG. In b), c), and d), the variations of the assumed causal relationships where the Assumption 1 is violated such that the traits of the individuals can influence the observed purchasing behavior through factors not related to friendship strength $S_{a,b}$.

The causal graph reflecting these assumptions is presented in Fig. 4. The illustrated graph is the standard DAG assumed to identify the causal effect of social influence under the presence of homophily in a pairwise setup when examining the causes behind why a person manifested a behavior at a given time (42, 43). The DAG is equivalent to the causal graph allowing for latent variables to influence both manifest network ties and manifest behaviors when the manifest behaviors are time-independent e.g. the choices are independent of each other, and there are no other unobservable biases.

According to backdoor criterion (62), the minimal sufficient adjustment set of variables for estimating the total effect of $Y_{a}(t)$ on $Y_{b}(t)$ is {$X_{a}$, P(t)}, therefore in our main analyses we match on partner’s identity to control for $X_{a}$ and common environmental factors to control for P(t). In Supplementary Material, Section S1.3 we consider how our estimation framework and the subsequent estimates vary as Assumption 1. is violated and additional controls are necessary.

Alternative methodological approaches, such as frequent itemset mining (63), can be used to extract co-occurring purchasing patterns. While these methods generally aim to identify associations in the data, related techniques like causal rule mining (64) enable rapid and accurate causal discovery. In the present study, we instead opted for a matched observational study design since our specific setting allows making causal assumptions. As a consequence, the matched inference does not face speed challenges inherent to multidimensional cause discovery.

### Matched estimation framework

####  

##### The setup

Given a partner *a* and a focal person *b*, let $Y_{a}(t)$ be the partner’s choice (set of purchased items within the transaction) and $Y_{b}(t)$ be the focal person’s choice (set of purchased items within the transaction). To estimate the total effect of the partner’s purchase on the focal person’s purchase ($Y_{a}(t)$ on $Y_{b}(t)$), we perform matched estimation. In Section ‘Causal assumptions and DAG’, given the assumed relationship between variables, the sufficient adjustment set of variables is the identity of the partner and the common environmental factors. Common environmental factors are operationalized by measuring the important dimensions of the dietary context: where the food is purchased (shop), when the food is purchased (time), the availability, and the popularity of the food, that date, that time of the day, in that shop as the fraction of all transactions that contained the food item. Availability at the purchase point is approximated by identifying what items were bought at least once for a given date, time of the day, and shop. We ensure that the same items were available.

##### Matching

We match dyads in order to find the matched pairs of comparable dyads where in one dyad partner buys the addition *i* ($i\in Y_{a}(t)$), whereas in the other partner does not buy the addition *i* ($i\notin Y_{a}(t)$). Within the matched pair of dyads, we ensure that the partner is the same person and that the dyads took place at the same shop and during the same time of the day (breakfast time vs. lunch time vs. afternoon/evening snack time). Additionally, we require that within the matched pair of dyads, the item was available in both dyads and equally popular (up to 10% caliper), and that both the focal person and the partner purchase the anchor item (meal or a beverage). The size of the popularity caliper was chosen to achieve the balance in covariates, before analyzing the outcomes.

##### Covariate balance

For all the covariates except food item popularity, an exact match is required. For popularity, we ensured that after matching standardized mean difference SMD<0.2 (before matching SMD=1.23, after matching SMD=0.08). Groups are considered balanced if all covariates have SMD<0.2, a criterion satisfied here (65).

##### Outcome analysis

After matching, we analyze 96,986 dyads, matched into 48,493 pairs of dyads. The distributions of dyads across additions are outlined in Supplementary Material, Table S1. The result is a set of matched pairs of comparable dyads, indistinguishable in the observed attributes, except that in one, the partner buys the additional food item, whereas in the other, the partner does not buy it. By focusing on different items, we apply our framework to measure the effect of different interventions, in different subpopulations. To quantify the effect of the exposure to the partner’s choice, our main analysis compares the purchases of the focal person in the matched pairs of dyads.

Given a food item *i*, $Y_{a}(t)$ partner’s choice (set of purchased items within the transaction) and $Y_{b}(t)$ focal person’s choice (set of purchased items within the transaction), we measure risk difference (${\mathrm{RD}}_{i}$) and risk ratio (${\mathrm{RR}}_{i}$) (66), calculated based on 2x2 contingency matrix, illustrated in Supplementary Material, Table S2. The two outcome statistics are defined as:


(1) $${\mathrm{RD}}_{i}=p(i\in Y_{b}(t)\;|\;i\in Y_{a}(t))-p(i\in Y_{b}(t)\;|\;i\notin Y_{a}(t)),\;\text{and}$$

(2) $${\mathrm{RR}}_{i}=p(i\in Y_{b}(t)\;|\;i\in Y_{a}(t))/\;p(i\in Y_{b}(t)\;|\;i\notin Y_{a}(t)).$$

The RD and RR describe the absolute and the relative difference in the observed risk of events between treated and control dyads. For a focal individual, they describe the estimated difference and the relative increase in the probability of purchasing the item. Within the comparable dyads, we resample to obtain the 95% CIs.

##### Ethical considerations

Nutrition is a potentially sensitive personal behavior. To protect user privacy, the log data used here was accessed exclusively by EPFL personnel involved in this project, and stored and processed exclusively on EPFL servers. The data were obtained with approval from EPFL’s Data Protection Officer and was anonymized before it was made available to the researchers for analysis.

## Supplementary Material

pgae517_Supplementary_Data

## Data Availability

Anonymized matched data are publicly available (67). Code necessary to reproduce the main findings is available at https://github.com/epfl-dlab/food-choice-mimicry.

## Preprints

This manuscript was posted on a preprint: https://arxiv.org/abs/2308.16095.
