# Supplementary material for: Food choice mimicry on a large university campus
Source: PNAS Nexus. 2024 Nov 16;3(12):pgae517. doi: 10.1093/pnasnexus/pgae517 (PMC11645252; doi:10.1093/pnasnexus/pgae517)
Supplement: pgae517_Supplementary_Data [file pgae517_supplementary_data.pdf]

Food Choice Mimicry on a Large University Campus: Supplementary Material

1.1 Supplementary information

We provide supplementary information and perform analyses that support our main conclusions or provide complementary insights. Figure S1 hows the histogram of dyads during a day and Figure S2 illustrates the effect across dyads, depending on the individuals status on campus. Figure S3 visualizes dose-response and sensitivity analysis results. Table S1 displays statistics about food addition items.

Table S2 shows the number of dyads in each condition depending on whether or not the partner purchased the item, and whether or not the focal person purchased the item. In Tables S3, S4, and S5, we present co-purchasing matrices that outline the dyad frequency among the subset of the studied dyads with demographic data available. The tables illustrate a preference for eating with others of the same gender, age, and status. In Fig. S4, we present the effect estimate among the subpopulation with demographic information available. The estimated risk difference across the matched pairs of dyads is shown separately, depending on the individuals' status, age, and gender.

In Fig. S5, we present the estimated risk difference across the nine years spanned by the purchase logs. Over the years, we find no notable trends in the estimated effect. Overall, across 13 food item additions, we measure the highest risk difference in 2011 (16.14% [14.25%, 18.05%]) and the smallest in 2016 (12.21% [10.73%, 13.67%]). In comparison, in the case of the randomized baseline, the highest risk difference is measured in 2018 (4.73% [3.35%, 6.13%]) and the smallest in 2016 (0.00% [-1.05%, 1.06%]). Consistently across years, the partner's influence on the focal person diminishes once the ordering of the queue is randomized. In Fig. S6, we present the relative version of the main findings by measuring the relative risk. Lastly, in Table S6 we list statistics about the product and shop variety, by status, and in Table S7, we list statistics about the top 20 most purchased food items.

1.2 Amplified asking

To make heterogeneous estimates depending on status at the campus (beyond the subpopulation of participants in the sustainability challenge), we rely on the paradigm of amplified asking to, first, build a model that can predict status in the sup-population where the status information is available, and then, second, amplify the entire dataset with the estimated class belonging, by making out-of-sample predictions over the whole population. We train the classifier based on the features that capture temporal patterns typical of personnel and staff. For instance, students make summer and winter breaks, while staff might still be on campus. Similarly, students might make transactions in the later hours.

We use the total number of transactions, the number of years at the campus, and the distribution of transactions across months, weekdays, and hours in the day. The classifier uses a random forest model. Two hyper-parameters (number of decision trees and number of splits in each decision tree were optimized). The classifier achieved, on a 20% held-out test set, a precision with respect to students of 88.33% and with respect to personnel of 78.26%, and a recall with respect to students of 90.60% and with respect to personnel of 76.60%. Note that status estimation does not rely on the variables linked with the studied phenomena (purchased items) but merely on the temporal distribution reflecting when the individuals are present on campus.

1.3 Robustness test: Effect estimate under different assumptions

First, we consider how our estimation framework and the subsequent estimates vary as Assumption 1 is violated. The alternative DAGs capture the relaxed assumptions. In Figure 4, Figures (b), (c), and (d), illustrate the variations of the assumed causal relationships where Assumption 1 is violated such that the traits of the individuals can influence the observed purchasing behavior through factors not related

to friendship strength $S_{a,b}$. For a set of plausible variations, we derive the minimal sufficient adjustment set of variables according to backdoor criterion. In particular:

1. Allowing partner's eating profile to influence the focal person's manifested behaviors through factors not related to friendship strength (Fig. 4b), the minimal sufficient adjustment set of variables for estimating the total effect of $Y_a(t)$ on $Y_b(t)$ is $\{X_a, P(t)\}$.
2. Allowing focal person's eating profile to influence partners manifested behaviors through factors not related to friendship strength (Fig. 4c), the minimal sufficient adjustment set of variables for estimating the total effect of $Y_a(t)$ on $Y_b(t)$ is $\{X_a, X_b, P(t)\}$.
3. Allowing both eating profiles to influence both manifested behaviors through factors not related to friendship strength (Fig. 4d), the minimal sufficient adjustment set of variables for estimating the total effect of $Y_a(t)$ on $Y_b(t)$ is $\{X_a, X_b, P(t)\}$.

Since the scenario depicted in Fig. 4 is already addressed by our main analysis, we investigate how robust estimates are when dyads are additionally matched on focal person identity to control for X_b (necessary in variations depicted in Fig. 4c and d. When additionally matching on focal person identity, we obtain the overall risk difference of 13.35% [12.82%, 13.89%], risk ratio of 1.76% [1.72%, 1.81%], and qualitatively similar findings as in our main analysis (cf. Fig. S8 for risk difference estimate across food items).

Second, we further investigate the impact of social tie strength. Social tie strength $S_{a,b}$ is operationalized by calculating the fraction of instances when the pair is eating together out of all instances when either one is observed eating with someone. In Fig. S7, we demonstrate that risk difference and risk ratio estimates are the greatest for the highest values of social tie strength. However, the estimates are significant in all strata of social tie strength, and, at minimum, focal persons are estimated to be +10% more likely to purchase the food item when the partner purchases vs. not.

1.4 Robustness test: Minimum number of transactions together

In the main analyses, we require that the two individuals make at least ten transactions together adjacent in the purchasing queues, to be able to observe the same pairs repeatedly. To test whether only people who know each other well buy similar foods is an alternative explanation for the results, we investigated the impact of this design choice.

Without this restriction, among dyads where $N < 10$, across all matched pairs, we obtain the overall positive risk difference of 5.54% [3.43%, 7.54%], risk ratio of 1.34% [1.22%, 1.51%], and qualitatively similar findings as in our main analysis for risk difference estimate across food items. Similarly, the partner's influence on the focal person entirely disappears (is not significantly different than zero) once the ordering of the queue is randomized (randomized baseline risk difference: 1.65% [-0.23%, 3.27%], risk ratio: 1.12 [0.99, 1.24]). These results imply that the effect is not limited only to people who know each other well and frequently eat together.

The fact that the effect is smaller among dyads with a fewer number of transactions together (compared to +14.22% increase in purchasing probability overall) is consistent with the finding that a high social tie strength is associated with the largest effects.

1.5 Robustness test: The impact of temporal order within a pair

Similarly, we performed a robustness test aiming to understand whether the temporal order of the focal person and partner eating together modulates the effect (i.e., whether, at a specific meal, the pair is observed eating together for the first, second, and up to the tenth time, limiting to pairs who have eaten together at least ten times). Using a least squares linear fit, we found no significant impact of the order on effect estimate, neither for risk difference ($\beta = 0.0012$, $p = 0.94$), nor for risk ratio ($\beta = 0.0013$, $p = 0.53$). However, we note that the largest effect is observed for the first instance of eating together ($RD = 16.14\%$ and $RR = 2.05$), which is consistent with the fact that nudge effectiveness is expected to be the strongest for the first exposure and decrease over time due to habituation.

1.6 Coordination hypothesis

An alternative hypothesis explaining the observed similarities between adjacent persons in the purchasing queue is that the two persons coordinated to go for a meal together and agreed on the food choice before lining up in the purchasing queue. We investigate the presence of such coordination.

There are 226 pairs of persons A and B such that there are at least ten matched pairs of dyads in order A–B and at least ten matched pairs of dyads in order B–A. For each pair, we independently test the coordination null hypothesis that the order A–B or B–A does not matter since similarities come from coordination before making a choice. Under the null hypothesis, people agree on what to eat together before lining up in the queue, so the order of how they go (A–B or B–A) does not make a difference. When the partner purchases an item, the focal person's probability of purchasing is the same in the two orders since the persons pre-agreed, i.e., the purchasing probability does not depend on the order.

Concretely, there is a set of matched pairs of dyads in A–B order and a set of matched pairs of dyads in B–A order. We calculate the purchasing probability of the focal person in the two sets and test the null hypothesis that they are the same. We pool across the 66 pairs by sampling the same number of dyads from each pair (ten), and then perform a two-sided t-test. We reject the coordination null hypothesis ($p = 3.9 \times 10^{16}$).

Based on this investigation, we conclude that it is unlikely that pre-purchase coordination can entirely explain the measured effect. Since dyads are matched, differences in ordering likely stem from different mimicry exhibited by person A and person B when they are the focal person vs. the partner.

1.7 Shop layout

In Fig. S9, we visualize the physical layout of the shops and mark the purchasing queues, food stations, and cash registries. Studied food item additions (such as a dessert or a condiment) are placed in font of the cash registry. However, the other food items offered in the shop can be stationed in various layouts (e.g., in a dedicated pasta station, or a salad-meal bar). We expect that shops with multiple cash registry stations display a lower mimicry effect, given that they potentially allow individuals who know each other to split and execute the purchase separately, at different cash registries.

Since shops vary in food offer, we monitor the estimated effect of mimicking the purchasing of pastry, the most frequent food addition item, available at all the shops (Table S1). We contrast two groups of shops—shops with a single cash registry (8 shops) and shops with two cash registries (4 shops). We measure significantly higher risk difference in shops with a single cash registry, compared to shops with two cash registries (16.77% [15.93%, 17.98%] vs. 13.72% [13.11%, 14.33%]). Furthermore, the four shops with the highest effect estimate are indeed shops with a single cash registry, where food stations tend to be arranged in a straight layout, in front of the cash registry (Fig. S9a). In summary, we find shops with multiple cash registry stations to have a lower estimated mimicry effect compared to shops with a single cash registry, as predicted.

1.8 Robustness test: Further controls

We also performed a robustness test additionally requiring that the matched pairs of dyads contain exactly the same anchor (meal vs. vegetarian mean; coffee vs. tea). This separate matching resulted in 94,664 matched pairs of dyads. This led to similar findings as in our main analysis (cf. Fig. S10 for risk difference and Fig. S11 for risk ratio estimates across food items).

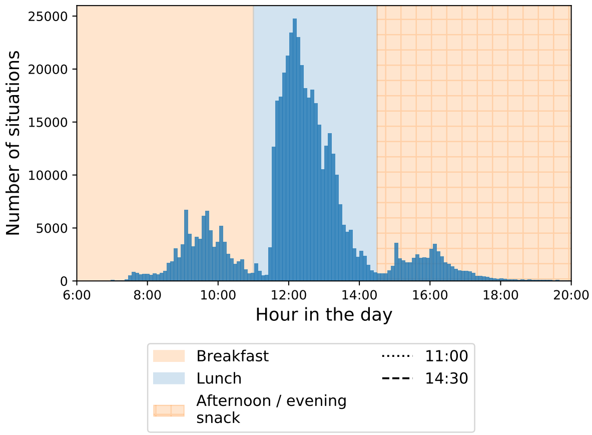

Figure S1: **The histogram of dyads during a day.** On the x-axis the hours in the day, and on the y-axis, the number of dyads. The three peaks correspond to breakfast, lunchtime, and afternoon or evening snack time (shaded regions).

Draft

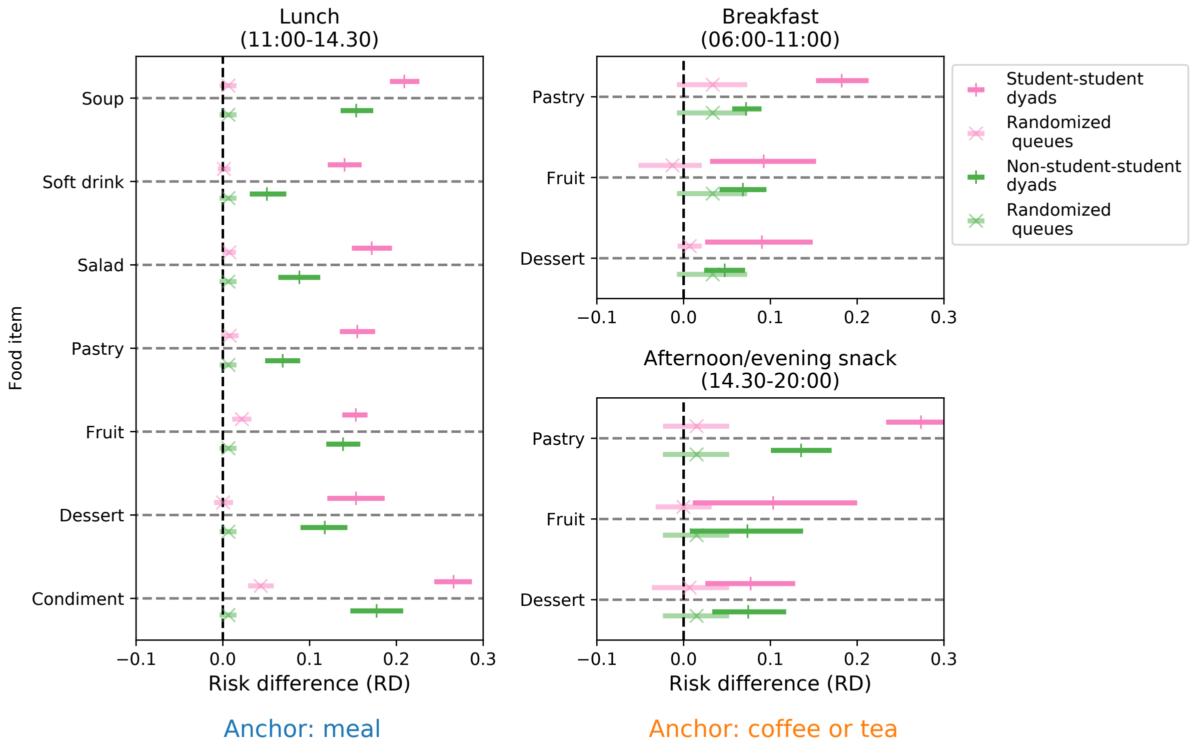

Figure S2: **Effect across dyads, depending on the individuals status on campus.** Separately for lunch, breakfast, and afternoon or evening snack, the estimated risk difference (on the x-axis), for the different food item additions (on the y-axis). The error bars mark 95% bootstrapped CI. Risk difference estimates are colored in pink for student-student dyads and in green for non-student–student dyads. Randomized baselines are presented in a lighter color.

Draft

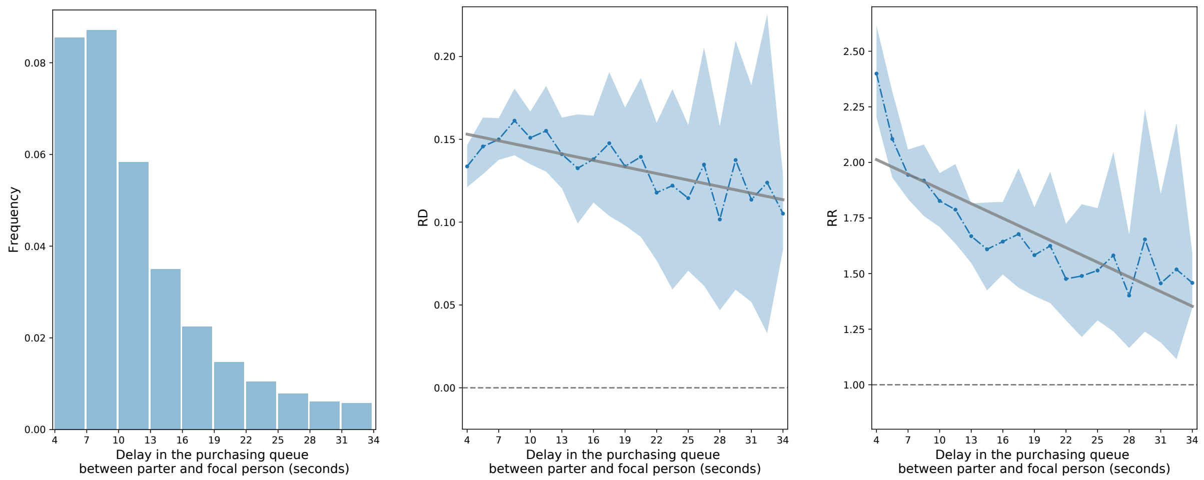

(a) Delay histogram (b) Risk difference (RD) (c) Risk ratio (RR)

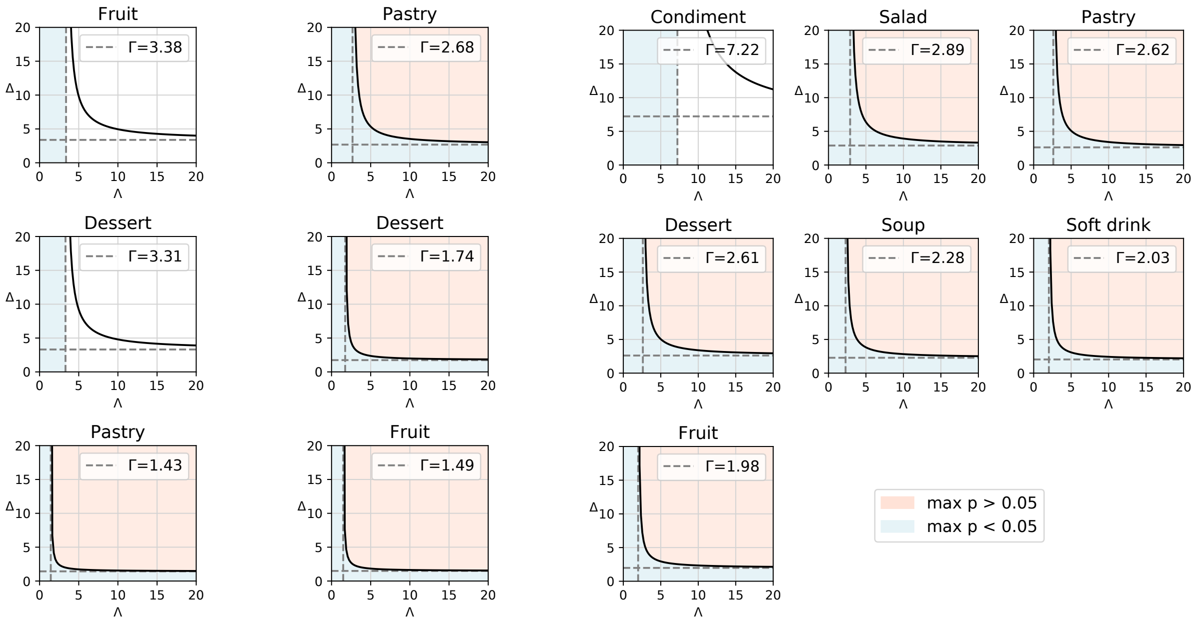

(d) Breakfast (e) Snack (f) Lunch

Figure S3: **Dose-response and sensitivity analysis.** In (a), the histogram of the temporal delay between the partner's and focal person's transactions among the dyads. On the x-axis, the delay, and on the y-axis, the frequency. In (b), the risk difference estimate within the subset of matched pairs of dyads (on the y-axis), with the given delay in the purchasing queue (on the x-axis). In (c), the risk ratio estimate within the subset of matched pairs of dyads (on the y-axis), with the given delay in the purchasing queue (on the x-axis). The shaded areas mark 95% bootstrapped CI. The gray dashed line represents the least square linear fit. Note the truncated x-axis; dyads with a delay of up to five minutes are considered, however they are rare as visible in (a). In (d), (e), and (f), sensitivity analysis. For the measured sensitivity Γ, the amplification (Λ, Δ) is plotted. Horizontal and vertical dashed lines indicate Γ, i.e., the asymptotic value of Λ for $\Delta \to \infty$, and vice versa.

Draft

Table S1: **Food addition item statistics.** For the three meals, the studied food addition items, the frequency with which the addition is purchased by the partner within the studied dyads (i.e., treatment frequency), and the number of matched pairs of dyads where the addition is purchased vs. not.

Time of day	Food addition item	Treatment freq.	# matched pairs of dyads
Breakfast/morning snack time	Dessert	8.77%	1004
	Fruit	3.62%	1226
	Pastry	7.85%	16898
Lunch time	Condiment	1.49%	5590
	Dessert	1.21%	3954
	Fruit	8.49%	22424
	Pastry	1.93%	7400
	Salad	1.51%	5286
	Soft drink	2.8%	8970
	Soup	7.79%	18956
Afternoon/evening snack time	Dessert	6.39%	1288
	Fruit	2.7%	466
	Pastry	16.54%	3524

Table S2: **Contingency table**. The number of dyads in each condition depending on whether or not the partner purchased the item (rows), and whether or not the focal person purchased the item (columns).

		Focal purchased		**Total**
		**No**	**Yes**	**dyads**
Partner	**No**	40230	8263	48493
purchased	**Yes**	33332	15161	48493
	**Total dyads**	73562	23424	**96986**

Table S3: **Gender co-purchasing matrix.** The condition frequency among the subset of the studied dyads with demographic data available. In rows, the gender of the focal person, in columns, the gender of the partner.

Partner<br>Focal person	Female	Male
Female	57.00%	10.65%
Male	12.67%	19.68%

Table S4: **Status co-purchasing matrix.** The condition frequency among the subset of the studied dyads with demographic data available. In rows, the status of the focal person, in columns, the status of the partner.

Partner<br>Focal person	Staff	Student
Staff	30.05%	9.63%
Student	9.83%	50.49%

Table S5: **Age co-purchasing matrix.** The condition frequency among the subset of the studied dyads with demographic data available. In rows, the age of the focal person, in columns, the age of the partner.

Partner<br>Focal person	≤22	23-32	>32
≤22	25.66%	2.34%	1.24%
23-32	2.67%	14.21%	9.45%
>32	0.02%	7.01%	37.40%

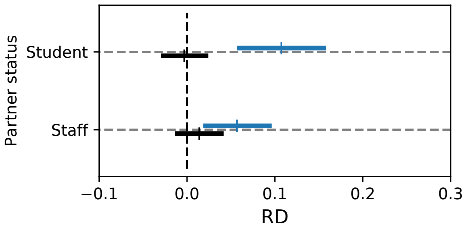

(a)

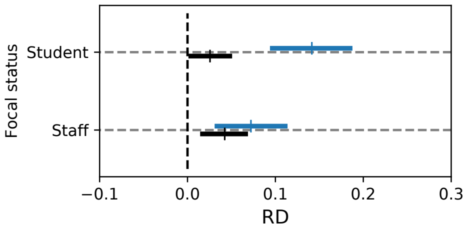

(b)

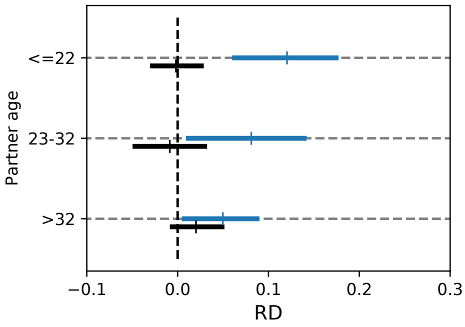

(c)

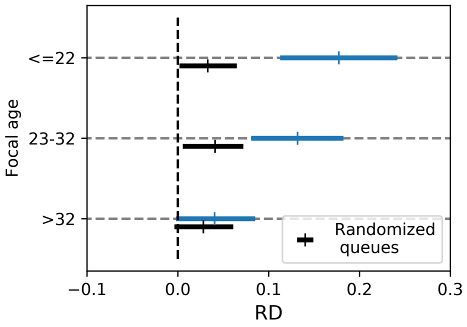

(d)

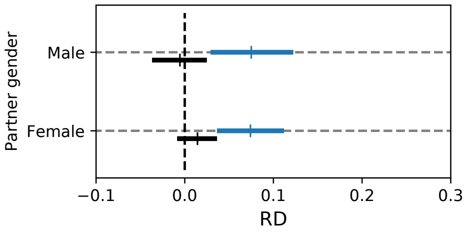

(e)

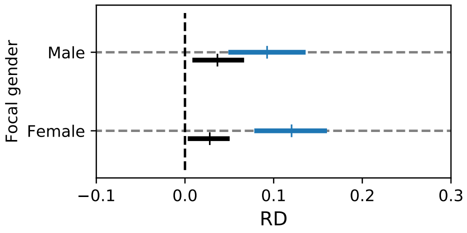

(f)

Figure S4: **Effect by status, age, and gender.** The estimated risk difference across the matched pairs of dyads (on the x-axis), depending on the individuals' status, age, and gender (on the y-axis). The error bars mark 95% bootstrapped CI. Risk difference estimates are presented in blue, the randomized baseline is presented in black. In (a) for partner's, in (b) for focal person's status, in (c) for partner's, in (d) for focal person's age, in (e) for partner's, and in (f) for focal person's gender.

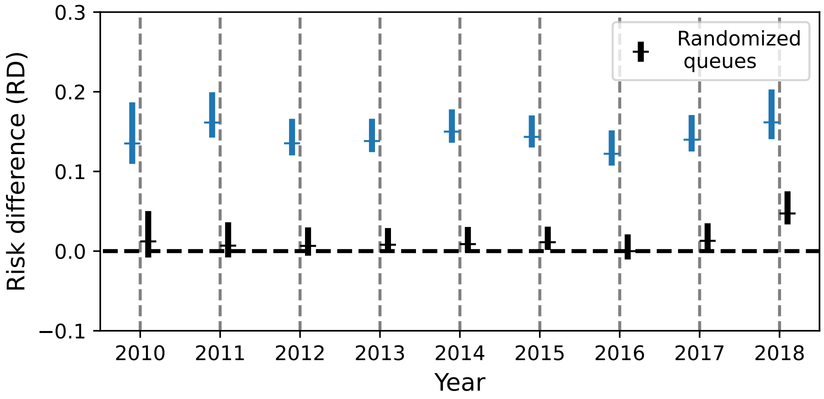

Figure S5: **Effect across the years.** Separately across years (on the x-axis), the estimated risk difference (on the y-axis). The error bars mark 95% bootstrapped CI. Randomized baseline is presented in black.

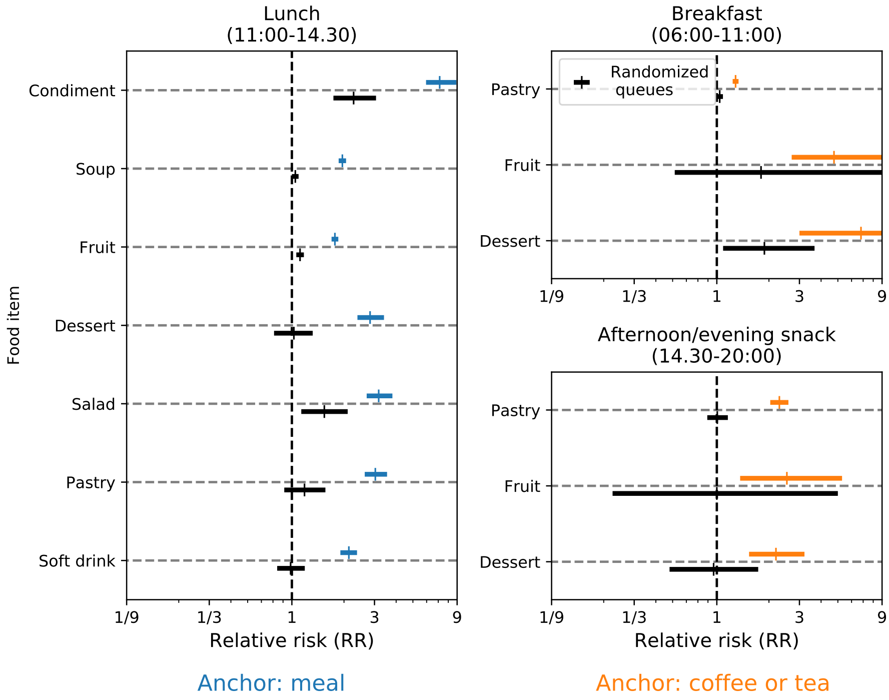

Figure S6: **Risk ratio estimates.** Separately for lunch, breakfast, and afternoon or evening snack, the estimated risk ratio (on the x-axis), for the different food item additions (on the y-axis). The error bars mark 95% bootstrapped CI. Relative risk estimates are colored (blue for lunch where the anchor is the meal, orange for breakfast and afternoon or evening snack where the anchor is a beverage). Randomized baseline is presented in black. Note the logarithmic x-axis.

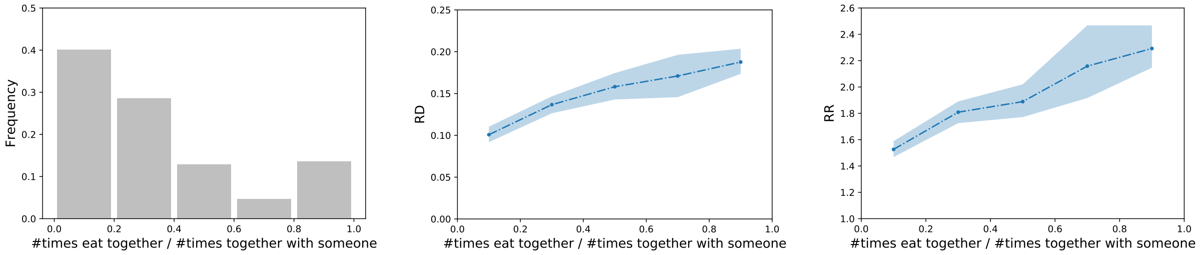

(a) (b) (c)

Figure S7: **The impact of the tie strength.** In (a), the histogram of the social tie strength between the focal person and the partner. In (b), the risk difference estimate within the subset of matched pairs of dyads (on the y-axis), with the given social tie strengths (on the x-axis). In (b), the risk ratio estimate within the subset of matched pairs of dyads (on the y-axis), with the given social tie strengths (on the x-axis). The shaded areas mark 95% bootstrapped CI.

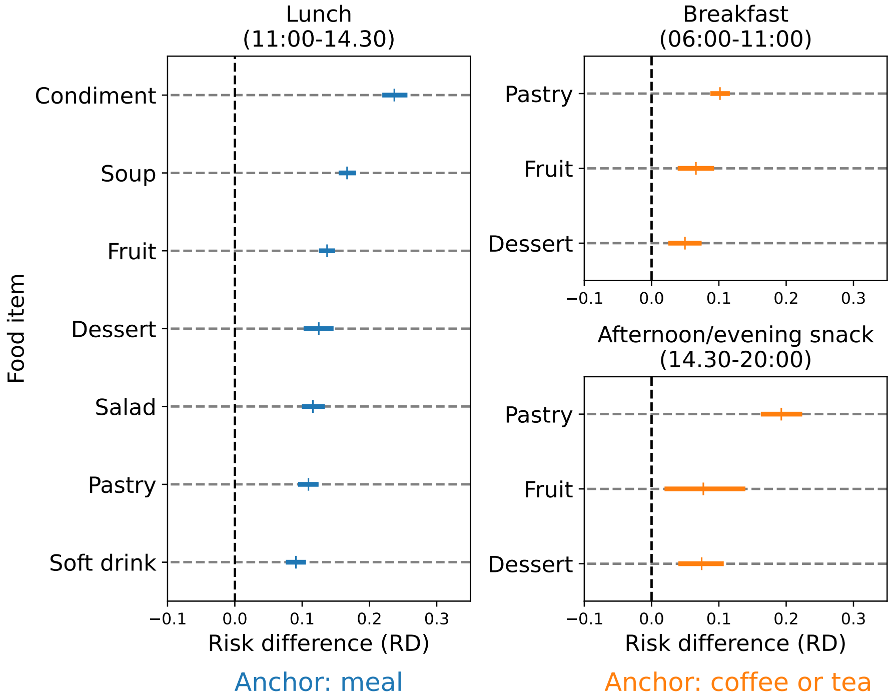

Figure S8: **Effect estimate under different assumptions.** Separately for lunch, breakfast, and afternoon or evening snack, the estimated risk difference (on the x-axis), for the different food item additions (on the y-axis). The error bars mark 95% bootstrapped CI. Risk difference estimates are colored (blue for lunch where the anchor is the meal, orange for breakfast and afternoon or evening snack where the anchor is a beverage).

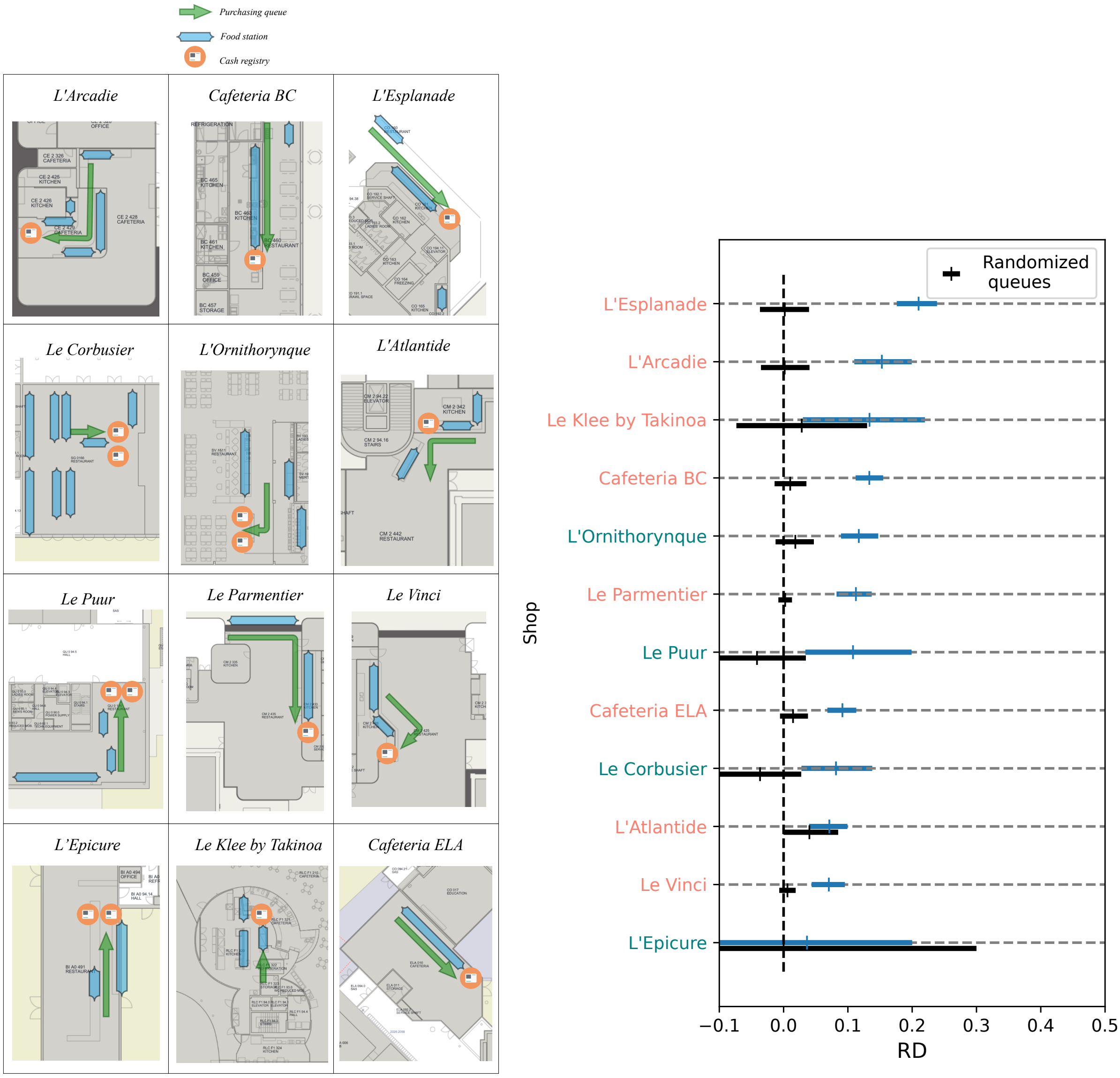

(a) (b)

Figure S9: **Shop layout.** In (a), visualization of the shop layout based on university map, with purchasing queues (green arrows), food stations (blue rectangles), and cash registries (orange circles) marked. In (b), the estimated risk difference for pastry addition, across the matched pairs of dyads (on the x-axis), across shops (on the y-axis). The error bars mark 95% bootstrapped CI. Risk difference estimates are presented in blue, the randomized baseline is presented in black. Orange shop names mark shops with a single cash registry, and blue marks shop names with two cash registries.

Draft

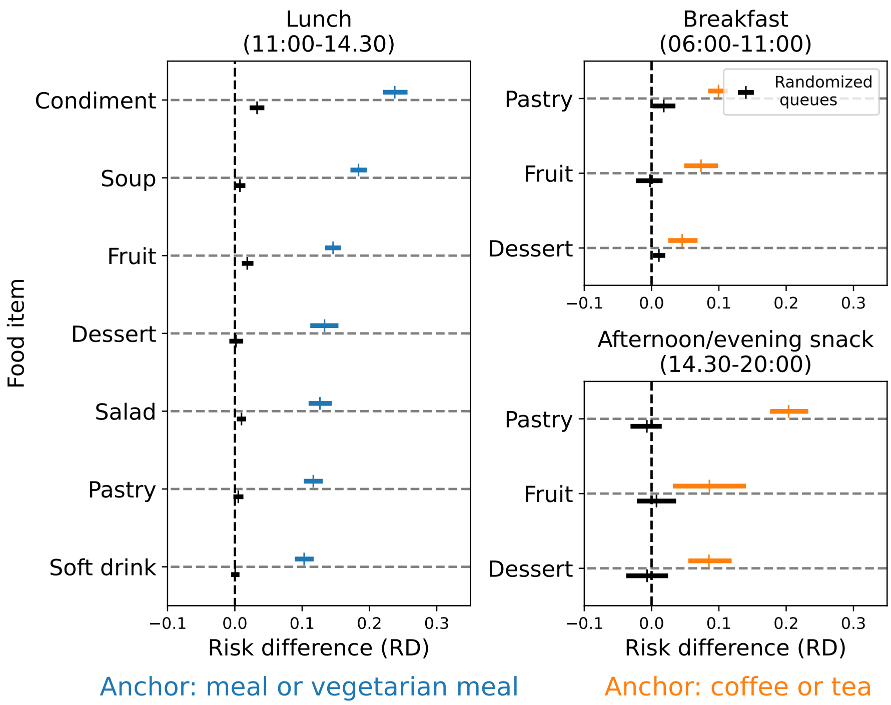

Figure S10: **Absolute effect estimate with additional anchor controls.** Separately for lunch, breakfast, and afternoon or evening snack, the estimated risk difference (on the x-axis), for the different food item additions (on the y-axis). The error bars mark 95% bootstrapped CI. Risk difference estimates are colored (blue for lunch where the anchor is the meal, orange for breakfast and afternoon or evening snack where the anchor is a beverage).

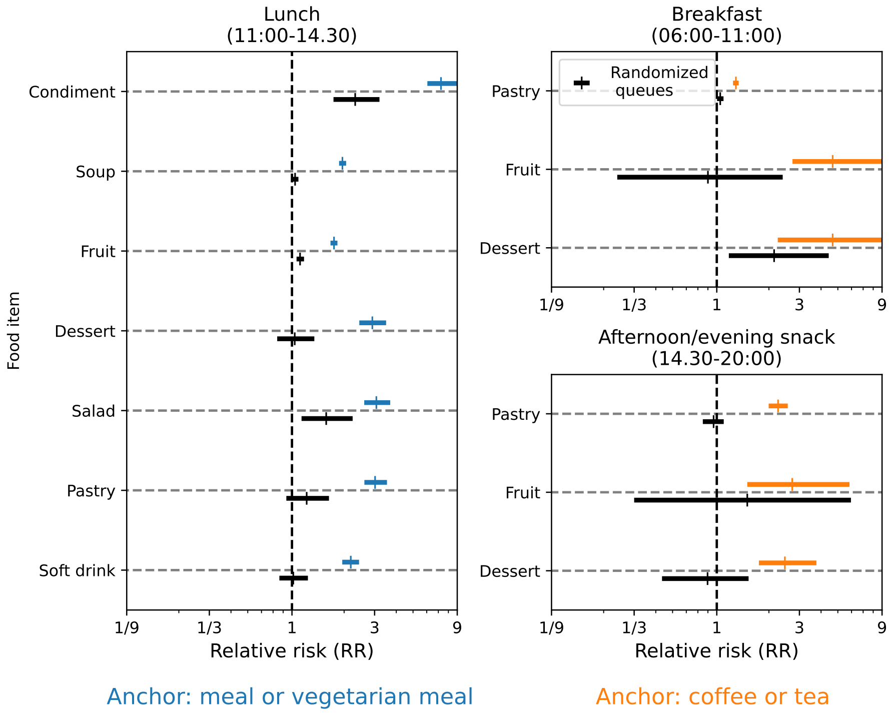

Figure S11: **Relative effect estimate with additional anchor controls.** Separately for lunch, breakfast, and afternoon or evening snack, the estimated risk ratio (on the x-axis), for the different food item additions (on the y-axis). The error bars mark 95% bootstrapped CI. Relative risk estimates are colored (blue for lunch where the anchor is the meal, orange for breakfast and afternoon or evening snack where the anchor is a beverage).

First k	Unique products per person		Unique shops per person	
transactions	Student	Staff	Student	Staff
10	M = 4.48, STD = 2.68	M = 4.47, STD = 2.67	M = 2.76, STD = 1.51	M = 2.06, STD = 1.16
20	M = 5.66, STD = 3.84	M = 5.65, STD = 4.05	M = 3.19, STD = 1.83	M = 2.35, STD = 1.48
30	M = 6.36, STD = 4.54	M = 6.10, STD = 4.84	M = 3.41, STD = 1.99	M = 2.43, STD = 1.56
40	M = 6.80, STD = 5.13	M = 6.41, STD = 5.25	M = 3.53, STD = 2.11	M = 2.49, STD = 1.60
50	M = 7.06, STD = 5.48	M = 6.63, STD = 5.47	M = 3.59, STD = 2.14	M = 2.55, STD = 1.62

Table S6: **Product and shop variety, by status.** For a given number of first executed transactions (k), the average number of unique products and the average number of unique shops per person. The statistics (mean and standard deviation) are displayed separately for students and staff members. Given the same number of executed transactions, students buy a larger number of different products and visit a larger number of shops than staff members.

Product name	Category	Shop	Number of transactions
Coffee	Coffee	Le Corbusier	363,778
Coffee	Coffee	L'Ornithorynque	315,621
Coffee	Coffee	Cafeteria ELA	282,097
Menu 1	Meal	Le Parmentier	279,916
Menu 1	Meal	Le Corbusier	268,898
Menu 1	Meal	Le Vinci	264,195
Menu 2	Meal	L'Ornithorynque	260,480
Menu 1	Meal	L'Ornithorynque	252,809
Menu 1	Meal	L'Atlantide	229,518
Menu 1	Meal	L'Esplanade	199,376
Coffee	Coffee	L'Esplanade	197,838
Cappuccino	Coffee	Le Corbusier	168,331
Coffee	Coffee	L'Arcadie	154,111
Menu 1	Meal	Cafeteria BC	150,304
Coffee	Coffee	Cafeteria BC	145,468
Coffee	Coffee	L'Atlantide	135,178
Menu 3	Meal	L'Ornithorynque	126,561
Coffee	Coffee	Le Klee by Takinoa	125,517
Menu 2	Meal	Le Parmentier	104,574
Chocolate croissant	Pastry	Le Corbusier	102,360

Table S7: Top 20 most purchased food items.
